# Call me by your name: Considerations of DNA sequences as types within wider discussions on fungal nomenclature

**DOI:** 10.1080/21501203.2023.2295412

**Published:** 2023-12-25

**Authors:** Nathan Edward Charles Smith

**Affiliations:** Lucy Cavendish College, University of Cambridge, Cambridge, UK

**Keywords:** Dark taxa, eponyms, Latin binomials, the International code of nomenclature (ICN)

## Abstract

This paper discusses the interaction between two substantial debates in taxonomy and nomenclature: The potential introduction of DNA-only types into fungal taxonomy and whether certain species names are offensive and should be changed. It argues that the acceptance of DNA sequences as types will likely lead to a proliferation of eponyms (species named after a person or persons) and that this will render them more likely to censure thus creating a point of instability in the fungal nomenclature. More fundamentally, it seeks to highlight the cultural and aesthetic attraction of names and to promote wider conversation on why we consider the Latin binomial central in our description of species.

## 1. DNA sequences as types

An area of mycology that has received a substantial amount of discussion over the past decade is the proposed move towards DNA sequences as types. To summarise a contentious area in brief, some mycologists seek to adapt the International Code of Nomenclature for algae, fungi, and plants (Turland et al. 2018) to allow for the description of new species without a physical type but instead from a DNA sequence – the number of which has increased substantially with metagenetics-based methodologies and which are currently referred to as the “dark taxa” [(Page 2016); an alternative term is the “dark matter fungi” (Grossart et al. 2016)]. The concept has been in discussion for over a decade (Hibbett et al. 2009, 2011) and was first formally proposed in 2016 (Hawksworth et al. 2016) and again in 2018 (Lücking et al. 2018). Whilst the initial proposals were voted down in both instances, the latter by substantial margins in the preliminary “guiding vote” (>85% of voters rejected both proposals; May and Miller 2018), the proposals were instead passed on to a special-purpose committee (Turland et al. 2017; May and Redhead 2018) and the general proposal remains prominent in the literature with the use of DNA sequences as types still considered favourably by some mycologists (Wu et al. 2019; Lücking et al. 2021; Nilsson et al. 2023).

This proposal has been criticised on a number of technical and disciplinary grounds (Thines et al. 2018; Zamora et al. 2018). One issue, however, that appears to have been undiscussed is how the names will be given to DNA-only types. The current advice for naming a species is guided primarily by convention bar requirements for ensuring the name is grammatically correct – even allowing “names that are arbitrarily formed” (Article 20.1). However, the general expectation is for the name to highlight its “unique, species-specific, attribute” about aspects such as country or region of origin, morphological characteristics, colour, or pattern (Rao 2022). When taxa are known only from DNA, given the lack of morphological characteristics, it is feasible that the origins of species epithets, generic names, and names of higher taxa, will come from three sources; names based on the characteristics of the DNA sequence, the location (both in terms of habitat and geography where the sample was collected), and, as has become an increasingly popular trend in species described from specimens across the wider biological taxa (Poulin et al. 2022; Mammola et al. 2023), after individuals – with this latter category known as eponyms. A fourth “source” of names, and perhaps the one likely to be the most abundant, would be alphanumeric names in the style of the genus *Darksidea* (Knapp et al. 2015; Romero-Jiménez et al. 2022), where the names of letters of the Greek alphabet have been utilised as specific epithets (such as *Darksidea phi*) – a naming convention that in and of itself provides a substantial challenge to the purpose and function of binomial names.

Previous attempts to go outside the mandate of the code to publish names based on DNA sequences as types (including types where the specimen is an environmental sample; Bridges and Hughes 2012; Kirk 2012; de Beer et al. 2016; Lücking and Moncada 2017; Khan et al. 2020) show similar proportions of naming traditions compared to species described from specimens in other taxa (Table 1), with only 2 of the 12 species described from DNA sequences being given eponymous epithets (although 9 of the 12 had eponymous genus names) compared to approximately 1/3 of recently described parasitic helminths (Poulin et al. 2022), ~2/10 of all spider epithets (Mammola et al. 2023), and ~ 3/10 of all *Aloe* L. epithets in use (Figueiredo and Smith 2010). However, the relatively small proportion of names associated with individuals is likely an effect of its limited application and the avant-garde and provocative nature of the papers, and, should DNA sequence types come to be accepted, it is reasonable to expect a proliferation of honorific or location-based naming given the lack of morphological characteristics available to describers. This presents a potential crisis for mycological taxonomy and the discussion presented here seeks to examine the potential impacts of the mass-emergence of eponymous names in mycology should proposed changes to the Nomenclatural Code be implemented.

**Table 1.** List of species and higher taxonomic described from DNA only including their entomologies.

DNA-only novel genera			
Name	Etymology	Etymology type	Reference
*Lawreymyces*	Dedicated to our esteemed colleague, James D. Lawrey, for his important contributions to lichen ecology and to our knowledge of lichenicolous fungi.	Named after Individual	Lücking and Moncada (2017)
DNA-only novel species			
name	Etymology of epithet.	Etymology type	Reference
*Lawreymyces bogotensis*	Growing in thalli of an unidentified *Normandina* collected within the capital district of Bogotá.	Named after location	Lücking and Moncada (2017)
*Lawreymyces columbiensis*	Growing on specimens of *Normandina columbiensis*	Named after habitat	Lücking and Moncada (2017)
*Lawreymyces confusus*	The epithet refers to the confusion caused by the first detected aberrant ITS sequence of this basidiomycete from a sample of *Normandina*.	Other	Lücking and Moncada (2017)
*Lawreymyces foliaceae*	Growing in thalli of *Agonimia foliacea*.	Named after habitat	Lücking and Moncada (2017)
*Lawreymyces palicei*	Named after Zdeněk Palice, the collector of the original material from which the sequences were obtained, and who first assessed the status of *Omphalina foliacea* as a species of *Agonimia* correctly.	Named after individual	Lücking and Moncada (2017)
*Lawreymyces pulchellae*	Growing in thalli of *Normandina pulchella*.	Named after habitat	Lücking and Moncada (2017)
*Lawreymyces spribillei*	This new species honours our colleague Toby Spribille, for his important contributions to our understanding of the lichen symbiosis and the discovery of the potential role of cryptic Basidiomycota in ascolichen symbioses.	Named after individual	Lücking and Moncada (2017)
*Hawksworthiomyces sequentia*	The epithet reflects the fact that this species is known only based on its DNA sequences.	Named after characteristic	de Beer et al. (2016)
*Archaeorhizomyces secundus*	At Ivantjärnsheden field station, the species is outnumbered for colonisation of the organic soil horizon by its closest known sister taxon *Archaeorhizomycetes victor*.	Named after characteristic	Khan et al. (2020)
*Archaeorhizomyces victor*	This species appear to win in competition with its closest known sister taxon for colonisation of organic soil at Ivantjärnsheden field station.	Named after characteristic	Khan et al. (2020)
*Piromyces cryptodigmaticus*	The epithet translates literally as “hidden specimen”.	Named after characteristic	Kirk (2012)
*Mortierella signyensis*	The epiphet comes from the location of the environmental sample.	Named after location	Bridges and Hughes (2012)

## 2. Current concerns around the use of eponyms

Much more than a mere aesthetic argument, recent activity in the broader fields of taxonomy and nomenclature have substantial implications for discussions of the naming of fungi, and particularly for the decision to allow DNA sequences as types. Recent research indicates that the etymology of a scientific name can influence scientific practice (Mlynarek et al. 2023), suggesting that eponyms may have a negative impact on promoting research into the species. Furthermore, the honorific naming of species has received criticism for having a substantial gender bias (Poulin et al. 2022; Vendetti 2022). Other, more pointed, discussions have centred broadly around whether certain species names are offensive or need changing to reflect indigenous or other knowledge systems (Gillman and Wright 2020; Knapp et al. 2020; Hammer and Thiele 2021; Smith and Figueiredo 2021, 2022; Thiele et al. 2022; Tracy 2022; Wright and Gillman 2022; Guedes et al. 2023). The majority of these criticisms have been explicit in their stated intention to amend the various nomenclatural codes. Whilst some arguments have limited themselves to specific words considered slurs in some languages (Smith and Figueiredo 2021), others have phrased the argument more broadly seeking to prioritise indigenous names (Gillman and Wright 2020; Wright and Gillman 2022), to remove names associated with individuals deemed to be “inappropriate and offensive” (Smith et al. 2022; Smith and Figueiredo 2022; Thiele et al. 2022; Tracy 2022; Roksandic et al. 2023), to reject names deemed “culturally offensive or inappropriate” (including those named in honour of a person that the taxonomic community agrees should not be honoured; Hammer and Thiele 2021), that eponyms should be restricted to honouring those with connection to biodiversity science (Smith and Figueiredo 2023), or to ban eponyms all together (Guedes et al. 2023; Mabele et al. 2023). The argument has also been applied to species bearing the names of historic regions deemed to have offensive names, such as Rhodesia (Smith and Figueiredo 2022). A number of critiques of these various approaches have been put forward, including the argument that it is a “slippery slope” to exclude some names, and questioning how it is possible to police the boundaries between acceptable and unacceptable eponyms (Mosyakin 2022a, 2022b; Mosyakin and Alford 2022; Ceríaco et al. 2023). The proposals have also been criticised on the basis that those proposing change are not representative of the groups who are affected by inappropriate names (Pethiyagoda 2023) If proposals to limit or exclude eponyms become accepted by the various nomenclatural codes, it is likely that naming after individuals will become more tenuous and seen as a point of instability in the nomenclatural code. This is because moral and ethical boundaries are unlikely to remain static and names that are considered acceptable now may be deemed unacceptable later (Mosyakin and Alford 2022; Mosyakin 2022b; Ceríaco et al. 2023). The current argument is that “some names that honour historical figures will be clearly appropriate, others will be clearly inappropriate, and others still will be in a ‘grey zone’ somewhere between these extremes” (Smith et al. 2022); a categorisation that provides no reassurances or answers to these questions and, instead, as has been previously highlighted, suggests a relativistic approach to acts of racism, other bigotries, and crimes, that will vary year on year depending on the political climate and those tasked with making the decision (Mosyakin and Alford 2022). In this, we might consider the prominent public figures who are currently honoured with species names. How will future taxonomists approach the polarising presidency of George Bush and the role of Dick Cheney (Abramowitz and Stone 2006; Abramowitz and Saunders 2008; Friedersdorf 2011; species descriptions: Miller and Wheeler 2005)? How will they interpret Beyonce’s connection to sweatshops (Mills 2016; Nissen 2016; species description: Lessard and Yeates 2011)? What will they make of the opinions and personal lives of John Cleese (species description: Thalmann and Geissmann 2005; Karasz 2019) and Leonardo DiCaprio (Stine 2022; species descriptions: Freitag et al. 2018; Gosline et al. 2022)? Perhaps, more pertinently, will they make a distinction in their moral assessment for species named after taxonomists and mycologists and species named after celebrities?

## 3. Discussion

Where does this leave the proposal to use DNA sequences as types? Whilst DNA-only types are not unique in the issues they face with naming – with many of the same argument also applying, albeit to a lesser extent, to cryptic species where physical types exists but the species are diagnosed solely on the basis of DNA differences – these issues are exasperated by the limitations of the approach. Due to a lack of characteristics, names assigned to DNA-only types are more likely to be eponymous than descriptive – particularly if naming is automated and conducted *en masse*. Indeed, tools for automated naming have already been developed – most notably by (Pallen et al. 2021), who, in response to the challenge of naming the next million bacteria and archaea, developed a programme, the “Great Automatic Nomenclator” (or Gan: https://github.com/telatin/gan), to generate a number of novel and grammatically-correct genus names. Importantly for this discussion, their work also encouraged the use of automated eponyms by creating a list of 80 “worthy scientists” and using this list to generate 640 potential genus names More broadly, such an approach provides its self-evident challenges to meaningful naming regardless of the origin of the type and is likely to further disconnect names from the species they posit to represent – particularly if names are generated “before they are tied to taxa”.

Recognising wider arguments occurring in taxonomy and nomenclature that seek to remove or alter species names – and particularly eponyms – based on concepts of decolonial practice and social justice, eponymous names are thus more likely to be unstable and provide complex issues to future taxonomists and mycologists. The wider arguments on the suitability of DNA types will probably continue and, as DNA technology becomes ever better more accessible, it will likely find more adherents – particularly if traditional skills in taxonomy continue to decrease (BBSRC and MRC 2014). Some of the technical arguments – particularly those relating to resolution and quality of sequences – will have less currency, though others focused on verification, reproducibility, and relatability are unlikely to be meaningfully answered with improvements in sequencing technology and storage. The issue of naming these species, however, will remain.

To take a wider view, we must recognise that the argument for applying Latin binomials to DNA sequence types extends beyond scientific arguments of classification and combinatorics (see Lücking 2019) and that Latin binomials have wider cultural and aesthetic value – in the sense that binomials are preferred, in part, because, compared to numerical or alphanumeric barcodes that have no standalone meaning, they are more attractive and more charismatic to mycologists and non-mycologists alike. Companies such as Taxon Expeditions (www.taxonexpeditions.com) base their business around the promise that customers will be able to name a new species. Indeed, the attractiveness of formal Latin binomials to the wider scientific community is explicitly recognised by some of the more prominent proponents of DNA-only types who admit that “informal names or numbers remain obscure without a broadly accepted, formal naming framework” (Lücking and Hawksworth 2018) and that formal names are necessary for dark taxa to be “taken seriously” (Nilsson et al. 2023). Yet it might equally be argued that such a formal naming framework will be weakened through the mass addition of less-descriptive names and, that the cultural value given to species through the process of assigning a formal Latin binomial is likely to be diluted should their names derive from increasingly tenuous links.

This is not to propose, as a recent paper has suggested (Guedes et al. 2023), that the *Code* should be modified to restrict the etymology of names available to mycologists (or anyone else). The naming of a species should always be at its discoverer’s discretion. Rather, this paper seeks to open up a wider discussion on why Latin binomials remain so central in discussing how to approach the analysis of undescribed species. Only in accepting the non-scientific attraction of Latin binomials can an honest conversation be had about how we treat the fungal “dark taxa” and the names we give to the things we cannot describe.
